# Volume of Disease as a Predictor for Clinical Outcomes in Patients With Melanoma Brain Metastases Treated With Stereotactic Radiosurgery and Immune Checkpoint Therapy

**DOI:** 10.3389/fonc.2021.794615

**Published:** 2022-01-12

**Authors:** Aidan M. Burke, Michael Carrasquilla, Walter C. Jean, Brian T. Collins, Amjad N. Anaizi, Michael B. Atkins, Geoffrey T. Gibney, Sean P. Collins

**Affiliations:** ^1^ Department of Radiation Oncology, Brody School of Medicine, East Carolina University, Greenville, NC, United States; ^2^ Department of Radiation Medicine, MedStar Georgetown University Hospital, Washington, DC, United States; ^3^ George Washington University Hospital, Washington, DC, United States; ^4^ Department of Neurosurgery, MedStar Georgetown University Hospital, Washington, DC, United States; ^5^ Georgetown Lombardi Comprehensive Cancer Center, Georgetown University, Washington, DC, United States

**Keywords:** SRS, brain metastases, immunotherapy, melanoma, radiation necrosis (RN)

## Abstract

**Purpose/Objectives:**

Clinical trials of anti-Programmed cell death protein 1 (PD-1) and cytotoxic T-lymphocyte-associated protein (CTLA-4) therapies have demonstrated a clinical benefit with low rates of neurologic adverse events in patients with melanoma brain metastases (MBMs). While the combined effect of these immunotherapies (ITs) and stereotactic radiosurgery (SRS) has yielded impressive results with regard to local control (LC) and overall survival (OS), it has also been associated with increased rates of radiation necrosis (RN) compared to historical series of SRS alone. We retrospectively reviewed patients treated with IT in combination with SRS to report on predictors of clinical outcomes.

**Materials and Methods:**

Patients were included if they had MBMs treated with SRS within 1 year of receiving anti-PD-1 and/or CTLA-4 therapy. Clinical outcomes including OS, LC, intracranial death (ID), and RN were correlated with type and timing of IT with SRS, radiation dose, total volume, and size and number of lesions treated.

**Results:**

Twenty-nine patients with 171 MBMs were treated between May 2012 and May 2018. Patients had a median of 5 lesions treated (median volume of 6.5 cm^3^) over a median of 2 courses of SRS. The median dose was 21 Gy. Most patients were treated with ipilimumab (n = 13) or nivolumab-ipilimumab (n = 10). Most patients underwent SRS concurrently or within 3 months of receiving immunotherapy (n = 21). Two-year OS and LC were 54.4% and 85.5%, respectively. In addition, 14% of patients developed RN; however, only 4.7% of the total treated lesions developed RN. The median time to development of RN was 9.5 months. Patients with an aggregate tumor volume >6.5 cm^3^ were found to be at increased risk of ID (p = 0.05) and RN (p = 0.03). There was no difference in OS, ID, or RN with regard to type of IT, timing of SRS and IT, number of SRS courses, SRS dose, or number of cumulative lesions treated.

**Conclusions:**

In our series, patients treated with SRS and IT for MBMs had excellent rates of OS and LC; however, patients with an aggregate tumor volume >6.5 cm^3^ were found to be at increased risk of ID and RN. Given the efficacy of combined anti-PD-1/CTLA-4 therapy for MBM management, further study of optimal selection criteria for the addition of SRS is warranted.

## Introduction

Melanoma is the fifth most common cancer in the USA with an estimated 106,110 new cases in 2021 (1). Approximately 13% of patients with melanoma present with brain metastases at diagnosis, and up to 40% of all patients with melanoma will develop brain metastases during the course of their illness (2). Progression of intracranial disease is a common cause of death once brain metastases are diagnosed, and historically, patients had a median survival of 6 months (3). Control of intracranial disease prolongs life and prevents neurologic morbidity. Surgery, radiotherapy, and radiosurgery have been investigated in the management of melanoma brain metastases (MBMs) either as single-modality treatments or in combination.

Whole-brain radiation therapy (WBRT) when added to stereotactic radiosurgery (SRS) has been shown to decrease distant brain recurrences in the setting of controlled systemic disease; however, it offers little, if any, survival advantage (4). Furthermore, WBRT has been shown in numerous studies of brain metastases to cause significant neurocognitive morbidity and decreased quality of life. More recently, SRS alone has become the standard of care for patients with brain metastases given the improved clinical and neurocognitive outcomes compared to WBRT (5, 6). SRS was initially reserved for patients with 1–3 brain metastases; however, a multi-institutional prospective observational study by Yamamoto et al. (7) demonstrated no difference in median survival or neurologic death-free survival between patients with 4 or fewer brain metastases and patients with 5–10 brain metastases. Furthermore, there was no difference in SRS-related complications. Given the propensity of melanoma to metastasize to the brain, this provides a rationale for aggressive treatment with SRS for multiple brain metastases.

One potential side effect of SRS is the development of radiation necrosis (RN). RN entails the temporary appearance of tumor enlargement with or without peritumoral edema. RN is often symptomatic with headaches or other focal neurologic deficits. Treatment for RN ranges from supportive to steroids, surgery, or bevacizumab. Approximately 50% of patients with RN improve at 6 months and 75% improve at 2 years (8). Previous analyses have investigated the association between lesion size and dose with regard to the incidence of RN. In the RTOG dose-escalation trial of single-fraction SRS following WBRT, the maximum dose with acceptable toxicity was found for lesion sizes <2 cm, 2–3 cm, and 3–4 cm. Notably, the rate of RN was found to increase over time without plateau, with a 2-year rate of 11% (9).

Prior to the advent of immune checkpoint inhibitors (ICIs), systemic therapy for metastatic melanoma was limited. In recent years, however, multiple ICIs such as CTLA-4 inhibitor ipilimumab and the PD-1 inhibitors nivolumab and pembrolizumab have been shown to be effective in the management of metastatic melanoma (10–14). In addition to their use for extracranial metastases, some of these agents have also been shown to have efficacy for the treatment of MBMs as monotherapy, combined therapy, or in combination with SRS (15–26). Improved outcomes have also been observed in patients receiving ICI and SRS for management of brain metastases with regard to local control (LC) and overall survival (OS). However, it has also been associated with increased rates of RN, with reported rates ranging from 21% to 37.5%, which is higher than those of historical studies of SRS alone (9, 27–31).

Given the high rates of LC and longer-term survival of patients with MBMs, it is important to better understand the risk factors for development of symptomatic RN following treatment with immune checkpoint therapy and SRS. Potential risk factors include type and timing of IT with SRS, size of lesions treated, number of lesions treated, total volume treated, and radiation dose. Herein we report our experience of SRS with ICI in patients with MBMs. We have analyzed the correlation between selected clinical variables and outcomes in our cohort with the objective of determining the rate of and factors predictive of RN in MBM patients treated with SRS and ICI.

## Methods

### Patients

With institutional review board approval, we reviewed all patients treated at Medstar Georgetown University Hospital with MBMs treated from 2013 to 2018 with SRS within 1 year of receiving systemic therapy with PD-1 and/or CTLA-4 inhibitors. Patients were included if they were treated for intact parenchymal brain metastases (n = 168, 98%) or postsurgical resection cavities (n = 3, 2%).

### Radiosurgery

All radiosurgical treatments were delivered with the CyberKnife (Accuray, Sunnyvale, CA, USA) stereotactic treatment system. The gross tumor volume (GTV) was delineated as the intact metastasis or the edge of the resection cavity, including all areas of contrast enhancement. There was no expansion for the planning target volume (PTV). Prescription dose, isodose line, and number of fractions were selected at the discretion of the radiation oncologist based on the size and number of lesions being treated. The volume of intracranial disease was calculated from treatment planning scans and is the same as the volume treated as reported by the Accuray treatment planning system.

### Immune Checkpoint Therapy

Nivolumab or pembrolizumab comprised the anti-PD-1 therapy regimen. Ipilimumab was used for anti-CTLA-4 treatment. Patients were treated with monotherapy or combined anti-PD-1/CTLA-4 therapy. The assigned regimen was the last course of systemic therapy prior to SRS in order to avoid the effects of multiple systemic agents influencing clinical end points. The date of immune checkpoint therapy given in closest proximity to each SRS treatment was used.

### Imaging

Multiplanar, multisequence MRI of the brain with and without gadolinium contrast was obtained prior to and at regular intervals following SRS. All patients underwent surveillance imaging every 2–3 months unless otherwise clinically indicated. Given the similarity of appearance of posttreatment effects such as pseudoprogression, tumor recurrence, and RN, careful attention was given to defining LC and RN. LC was defined as freedom from progression in a previously treated lesion requiring repeat radiosurgery, neurosurgical intervention, permanent neurologic symptoms, or death. RN was defined as having both radiographic and symptomatic evidence of RN. Radiographic evidence of RN was defined as new enhancement or changes in treated lesion read as concerning for RN by neuroradiology that remained stable or resolved on subsequent imaging without further surgery or radiation.

### Statistical Analysis

The Kaplan–Meier method was used to calculate OS, LC, and freedom from RN from start date of radiation until death, progression, or RN, respectively. Log-rank analysis was used to compare groups. Cox regression analysis was used to identify factors associated with increased risk of RN or worse OS. SPSS Statistical Software (IBM Corporation, Armonk, NY, USA) was used for all statistical analyses.

## Results

### Patient and Treatment Characteristics

A total of 29 patients with 171 MBMs were treated with ICI and SRS between 2013 and 2018. Median age was 62, and all patients had an Eastern Cooperative Oncology Group (ECOG) performance status of 0 or 1. Patients were treated with ipilimumab (n = 13), ipilimumab and nivolumab (n = 10), pembrolizumab (n = 5), and pembrolizumab and ipilimumab (n = 2). The majority of patients (n = 21) received SRS concurrently or within 30 days of ICI, while a few (n = 8) were treated with SRS more than 30 days prior to or following ICI. Specific patient characteristics are shown in **Table 1**.

**Table 1:** Patient characteristics.

Patients	n (%)
Total patients	29 (100)
Age <60	13 (45)
Age >60	16 (55)
ECOG Performance Status	
0	13 (45)
1	16 (55)
Immunotherapy	
lpilimumab	13 (45)
lpilimumab + Nivolumab	10 (35)
lpilimumab + Pembrolizumab	5 (17)
Pembrolizumab	2 (3)
Timing SRS + IT	
Concurrently	21 (72)
Sequentially	8 (18)

### Radiotherapy Treatment Characteristics

A total of 171 MBMs were treated with a median volume of intact metastases of 0.44 cm^3^ (range 0.04–13.61 cm^3^). Patients were treated in one course (n = 12), two courses (n = 11), or more than two courses (n = 6, range 3–7). The majority of lesions were treated with a single fraction (n = 160/171 lesions, 93.5%) to a median dose of 2,100 cGy (range 800–2,100 cGy). Patients were cumulatively treated to a single lesion (n = 7), two to five lesions (n = 6), or greater than five lesions (n = 16, range 6–23). The median cumulative volume treated was 6.5 cm^3^ (range 0.2–22 cm^3^). Treatment characteristics are shown in **Table 2**.

**Table 2:** Treatment characteristics.

Lesions	n (%)
Total lesions	171
Volume of intact lesions (em^3^)	
Median (range)	0.44 (0.04–13.61)
<0.1	13 (8)
0.10 - < 0.3	40 (23)
0.30 - < 1.0	43 (25)
1.00 - < 2.0	20 (12)
2.00 - < 5.0	17 (10)
>5.0	11 (6)
n/a	27 (16)
Total volume treated (cm^3^)	
Median volume	6.5
<6.5	14 (48)
>6.5	15 (52)
No. of fractions (median dose, range)	
1 (2,100, 800–2,100)	160 (93)
3 (2,700, 2,400–2,700)	6 (3)
4 (2,700, 2,700–3,200)	3 (2)
5 (3,000, 3,000–3,000)	2 (2)
SRS prescription isodose line, median (range)	88 (75–95)
Cumulative number of courses	
1	12 (41)
2	11 (38)
>3	6 (21)
Number of lesions treated	
1	7 (24)
2–5	6 (60)
>5	16 (56)

### Clinical Outcomes

**Figure 1:**
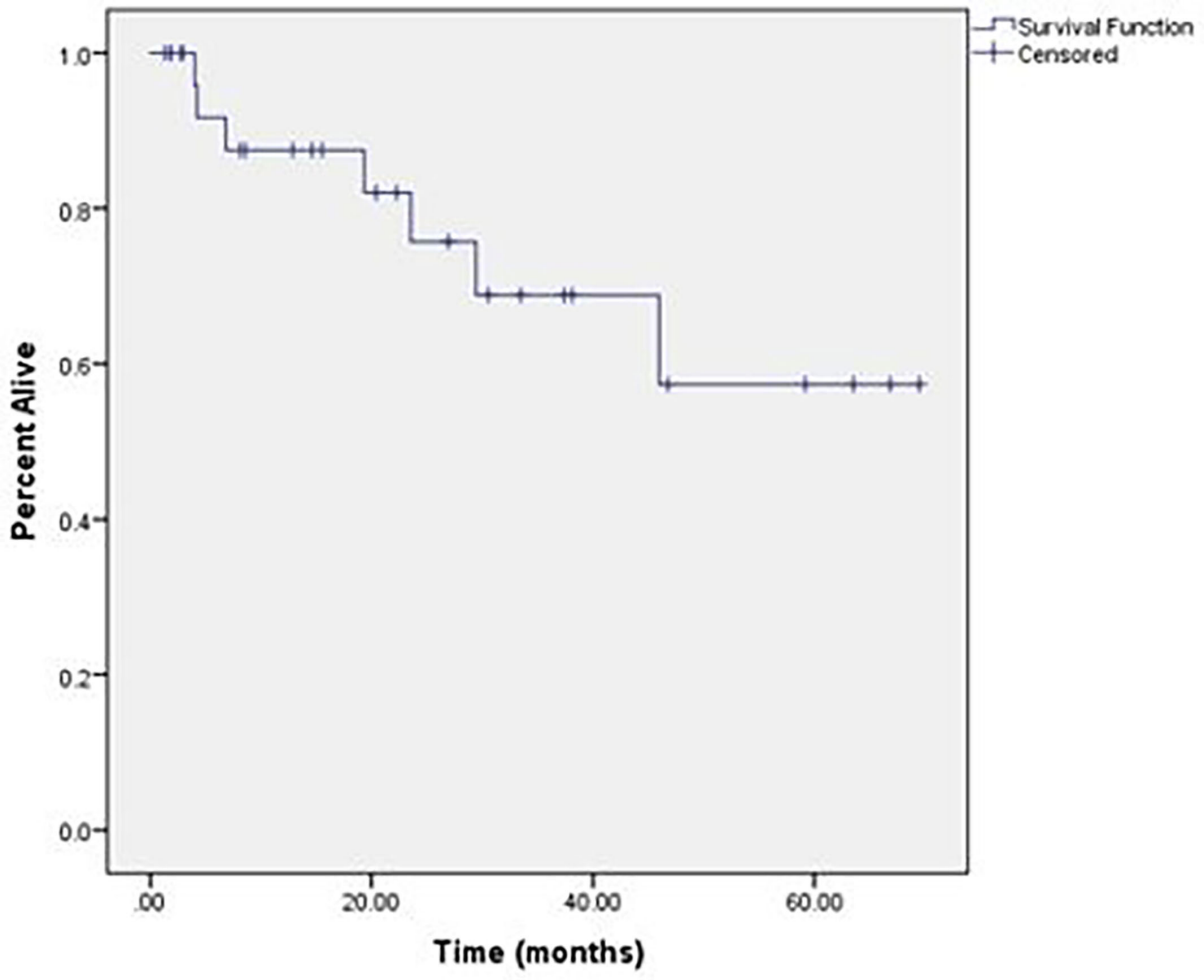
Local control of all treated lesions.

**Figure 2:**
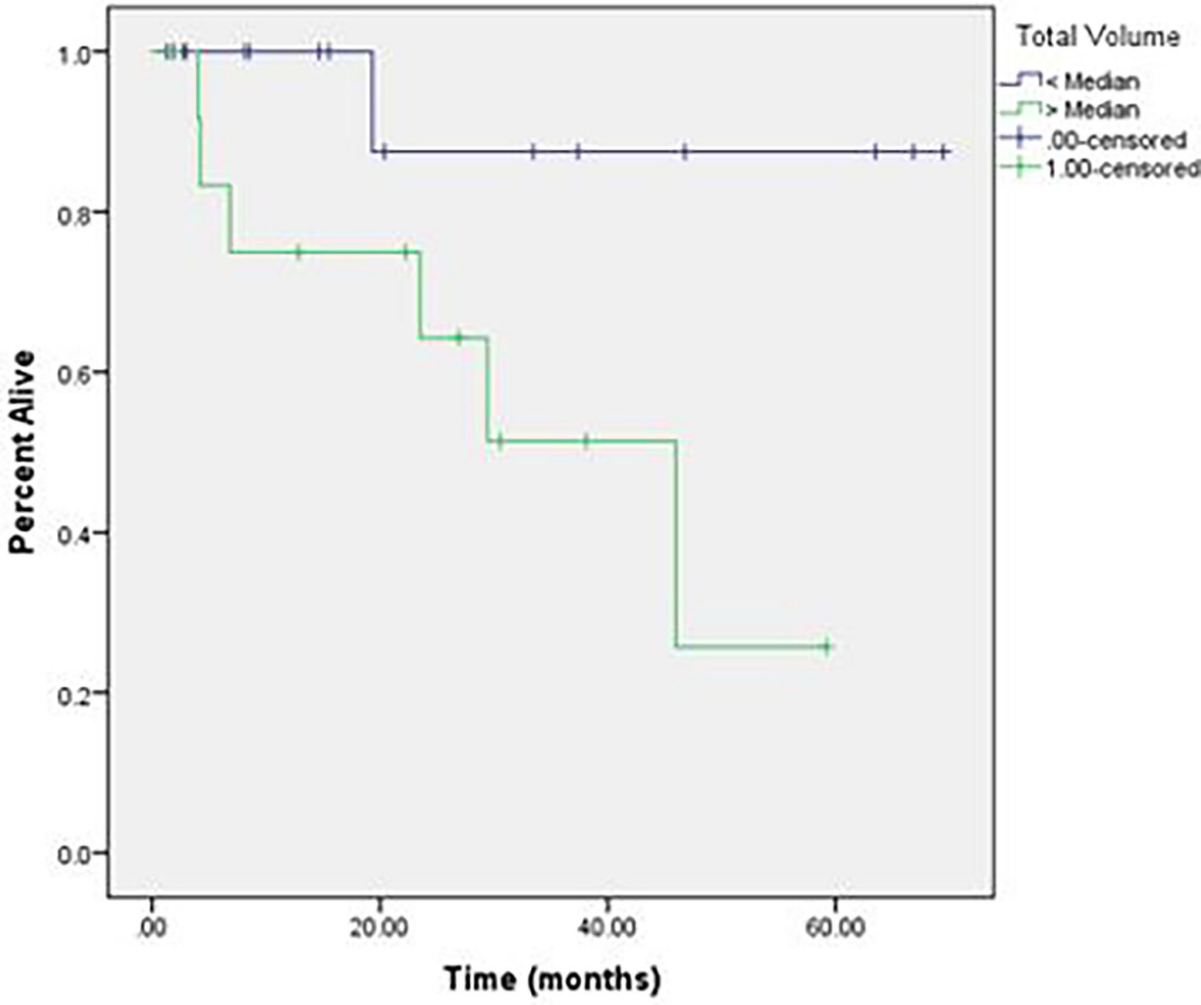
Intracranial death in patients treated to cumulative volume > 6.5 cm^3^ vs < 6.5 cm^3^.

**Figure 3:**
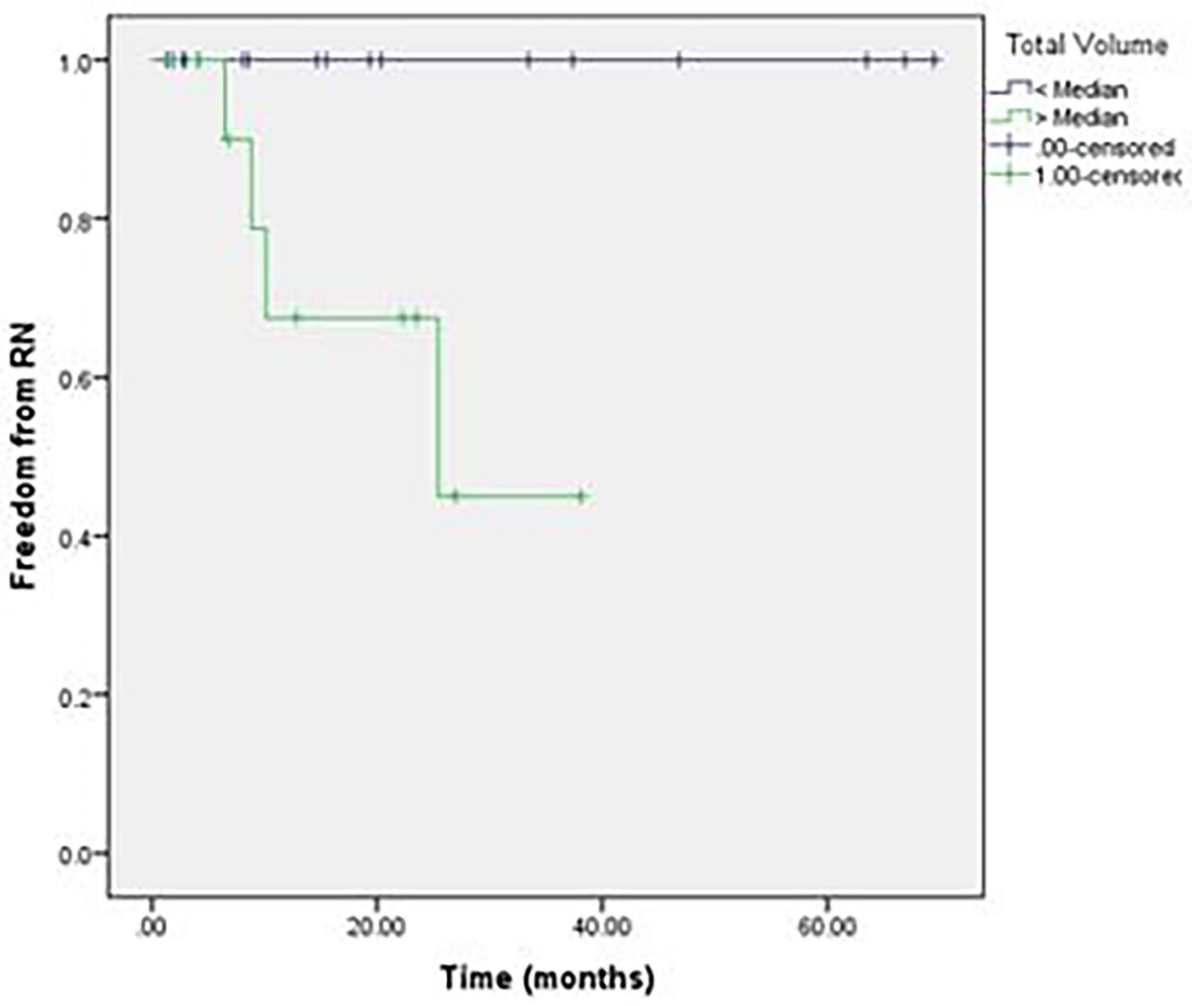
Radiation necrosis in patients treated to cumulative volume >6.5 cm^3^ vs < 6.5 cm^3^.

With a median duration of follow-up of 20.4 months, the 2-year OS was 55% and median OS was 29.4 months, with only 25% of deaths occurring from intracranial disease. The intracranial LC at 2 years was 76% (**Figure 1**). Overall, 14% of patients developed RN; however, only 4.7% of the total number of treated lesions developed RN. More than half of the patients (59%) required more than one course of SRS secondary to development of new brain metastases following initial SRS. The median volume of treated intracranial disease was 6.5 cm^3^, and the median number of lesions treated was 5. Patients who were treated to >6.5 cm^3^ aggregate volume were at an increased risk of intracranial death (log-rank, p = 0.046) and RN (log-rank, p = 0.027) compared to those treated to smaller volumes (**Figures 2**, **3**, respectively). Other variables, including number of lesions treated, number of courses, initial drug treatment, monotherapy vs. combination ICI therapy, and SRS within 30 days of ICI, were not significantly associated with clinical outcomes.

## Discussion

As ICI and SRS have become the mainstay of treatment for MBMs, it is important to better understand their combined effects on both tumor and normal tissue. In our institutional analysis of patients with MBMs treated with SRS and immune checkpoint therapy, we noted high rates of LC for treated lesions, with approximately 60% of patients requiring additional SRS for new brain metastases following their initial treatment. Overall, 14% of patients developed symptomatic RN following SRS. Patients with increased volume of treated brain metastases had significantly worse outcomes with regard to development of RN and death from intracranial progression compared to patients with a smaller burden of disease. Other variables, including total number of brain metastases, were not significantly associated with rate of RN or death from intracranial progression.

Numerous studies have investigated combined SRS with immune checkpoint therapy with reported rates of RN between 2% and 37.5% (28). Colaco et al. (27) found that the RN rate was increased significantly in patients who received ICI compared to chemotherapy (25.5% vs. 16.9%, respectively). Similarly, Fang et al. (28) found that 27% of patients developed RN when treated with gamma knife SRS and ICI. There was no clear association of ICI type or temporal proximity to SRS with increased risk of RN; however, treating an increased number of lesions or larger lesions in a single session was associated with increased risk of RN (28). In their series of 57 patients, Patel et al. (16) did not, however, find a significant difference in the rates of RN in patients treated with ipilimumab and SRS compared with SRS alone (30% vs. 21%, respectively, p = 0.08). A recent report of ICI and Non-small cell lung cancer (NSCLC), renal cell, and MBMs treated with SRS showed an association between ICI and development of symptomatic RN, especially in melanoma (30). In their report on long-term neurotoxicity of SRS + ICI in patients with MBM, Da Silva et al. (32) found that 18% of patients (21 of 118) developed RN. They concluded that RN is a significant toxicity in melanoma patients with brain metastases treated with ICI and SRS, particularly in long-term survivors.

In our study, we found a similar overall rate of RN of 16%; however, when accounting for number of lesions treated, only 4.7% of all metastases treated developed RN. We did not find any association between type of ICI or timing of ICI and SRS with regard to development of RN. Patients with a median volume of treated brain metastases greater than the median value of 6.5 cm^3^ volume were at increased risk of intracranial death (log-rank, p = 0.046) and RN (log-rank, p = 0.027) compared to those treated to smaller volumes. With a median duration of follow-up less than 2 years, these numbers may continue to increase over time, although most reports demonstrate a median time to RN of 9–12 months (17, 27, 32). Given the overall 2-year rate of LC with only one patient in our series experiencing a local failure, it is possible that lower doses of radiation could be used to achieve acceptable rates of LC while decreasing the risk of RN.

In the recently reported phase II trial Checkmates 204, patients with MBMs <3 cm and without neurologic symptoms or requiring local intervention were treated with ipilimumab and nivolumab with the primary end point of rate of intracranial clinical benefit. In their study of 94 patients, 26% had a complete response and 30% had a partial response in the brain, yielding a new response rate of 55%, with only 2% of patients receiving stereotactic radiotherapy during the study. Grade 3 or 4 toxicity was observed in 55% of patients, the most common being hepatotoxicity, and overall, 20% of patients had to discontinue treatment because of an adverse grade 3 or 4 event. Intracranial progression-free survival was observed in 60% of patients at 12 months from start of treatment (26). These results are further substantiated by another recently reported phase II study of a similar patient population treated with nivolumab monotherapy vs. ipilimumab combined with nivolumab (25). The study showed an intracranial response rate of 56% and 6-month progression-free survival rate of 53% in treatment-naive MBM patients treated with nivolumab plus ipilimumab.

Given the intracranial clinical activity of nivolumab plus ipilimumab and the potential for RN with SRS and ICIs, asymptomatic patients with MBMs <3 cm are now generally offered up front nivolumab plus ipilimumab and monitored closely with serial brain imaging. However, in symptomatic patients, particularly those with corticosteroid requirements or those with intracranial disease progression on nivolumab plus ipilimumab, SRS remains a standard of care. Our current dose of 21 Gy was based partially on the relative radioresistance of melanoma as well as the results reported in RTOG 95-05 that looked at single-fraction doses of recurrent, previously irradiated primary brain tumors and metastases (9). Using doses >20 Gy for small lesions is generally considered safe; however, in the setting of treatment with ICI, lower doses may prove to be as effective and carry less risk for developing RN. Limitations of this study include its relative small sample size, low event rate, which may limit our ability to identify correlations, as well as the limitations inherent to any retrospective analysis. As clinical outcomes continue to improve in this patient setting, continued research is needed to determine the ideal sequencing and dosing of systemic agents and SRS.

## Data Availability Statement

The raw data supporting the conclusions of this article will be made available by the authors without undue reservation.

## Ethics Statement

The studies involving human participants were reviewed and approved by Georgetown University Hospital IRB2017-1149. Written informed consent for participation was not required for this study in accordance with the national legislation and the institutional requirements.

## Author Contributions

This study was not supported by outside funding. AB, BC, MC, and SC all contributed to study concept, design, and/or acquisition of data. AB and MC completed the data collection. MC, AB, and SC contributed to the data analysis. MC, AB, SC, BC, MA, GG, AA, and WJ were responsible for drafting the article. All authors contributed to revising and giving final approval to the article. All authors agree to be accountable for all aspects of the work including its accuracy and integrity.

## Conflict of Interest

The authors declare that the research was conducted in the absence of any commercial or financial relationships that could be construed as a potential conflict of interest.

## Publisher’s Note

All claims expressed in this article are solely those of the authors and do not necessarily represent those of their affiliated organizations, or those of the publisher, the editors and the reviewers. Any product that may be evaluated in this article, or claim that may be made by its manufacturer, is not guaranteed or endorsed by the publisher.
